# Development and characterization of gelatin–platelet-rich plasma hydrogels for enhanced spinal cord injury repair

**DOI:** 10.1038/s41598-026-49004-1

**Published:** 2026-04-14

**Authors:** Shima Ghiasi Moradi, Hesam-Uddin Hoseinzadeh, Hamed Rad

**Affiliations:** 1 Department of Clinical Sciences, Ka. C., Islamic Azad University, Karaj, Iran; 2 Department of Pathobiology, Ka. C., Islamic Azad University, Karaj, Iran

**Keywords:** Spinal cord injury, Platelet-rich plasma, Gelatin hydrogel, Biomaterials, Neural regeneration, Biotechnology, Medical research, Neuroscience

## Abstract

Spinal cord injury (SCI) remains a major clinical challenge due to the limited regenerative capacity of the central nervous system. This study presents the development and characterization of a gelatin–platelet-rich plasma (PRP) hydrogel as a therapeutic strategy for SCI repair. A composite gelatin–PRP scaffold was fabricated by incorporating PRP into a gelatin matrix to enhance tissue regeneration and functional recovery. In vitro analyses demonstrated excellent biocompatibility, with neuronal cell viability increasing from 75% on Day 1 to 96% on Day 7 (*p* < 0.05). The release of growth factors including transforming growth factor β1 (TGF-β1), platelet-derived growth factor BB (PDGF-BB), and insulin-like growth factor 1 (IGF-1) followed a biphasic pattern, with approximately 85–88% released within 96 h (*p* < 0.05). In vivo, the PRP + Hydrogel group exhibited significantly improved locomotor recovery on the Basso–Beattie–Bresnahan (BBB) scale, along with reduced cavity formation, improved tissue preservation, and decreased scar-like tissue appearance compared with other treatments (n = 6 per group, *p* < 0.05). These findings indicate that gelatin–PRP hydrogels support growth factor delivery, promote tissue repair, and improve functional recovery, highlighting their potential as a biomaterial platform for spinal cord repair.

## Introduction

Spinal cord injury (SCI) is a debilitating neurological condition that affects millions of people worldwide, often resulting in permanent disability and a profound reduction in quality of life. Each year, approximately 130,000 new cases are reported globally, many of which lead to life-altering outcomes such as paralysis, sensory deficits, and impaired motor function^1^. Beyond the individual burden, SCI imposes significant emotional, social, and economic challenges on families and healthcare systems. Despite advances in neurobiology and clinical management, effective treatment of SCI remains elusive due to the limited regenerative capacity of the central nervous system (CNS)^2–4^. Unlike peripheral nerves, which possess some intrinsic ability to repair, the spinal cord exhibits minimal regenerative potential, leaving most injuries largely irreversible^5^.

The pathophysiology of SCI involves a cascade of primary and secondary injury processes that collectively hinder neural repair. The initial mechanical insult disrupts axons and blood vessels, causing hemorrhage, ischemia, and inflammation. This is followed by oxidative stress, excitotoxicity, and the activation of glial cells, all of which exacerbate neuronal death and tissue damage^2,3,6^. One of the most significant barriers to recovery is the formation of a dense glial scar, which physically obstructs axonal regrowth and creates a non-permissive microenvironment for regeneration^3,7,8^. Consequently, conventional therapeutic strategies including surgical decompression, pharmacological interventions, and stem-cell-based therapies have achieved only limited success in restoring motor or sensory function^4,9^. These limitations underscore the urgent need for innovative biomaterial-based approaches that can overcome the intrinsic barriers to CNS regeneration.

In recent years, biomaterials have emerged as a promising frontier for SCI treatment, particularly through tissue-engineering strategies^10–12^. Biomaterials can function as scaffolds that support cellular adhesion, migration, and survival, thereby promoting tissue repair and structural stability at the lesion site. Among these, hydrogels are especially attractive due to their high water content, biocompatibility, and structural similarity to the extracellular matrix (ECM)^13–15^. Although the biochemical and structural complexity of natural ECM is greater than that of synthetic or semi-synthetic hydrogels, hydrogels can mimic several key physical properties of the ECM and serve as supportive scaffolds for tissue regeneration when combined with bioactive molecules^16–18^. Their porous, hydrated architecture enables efficient infiltration of cells and diffusion of nutrients, while their tunable mechanical properties allow adaptation to specific tissue requirements^11,19^. Moreover, hydrogels can be engineered to incorporate bioactive molecules such as growth factors or cytokines, enabling controlled and sustained release at the site of injury^14–16^. In SCI models, hydrogels provide a permissive environment for axonal growth, neural survival, and tissue regeneration, making them strong candidates for translational neurorepair applications^20^.

Another emerging therapeutic strategy is the use of platelet-rich plasma (PRP), an autologous blood derivative enriched in growth factors and cytokines that promote cellular proliferation and angiogenesis^21^. Key bioactive mediators including platelet-derived growth factor-BB (PDGF-BB), transforming growth factor-β1 (TGF-β1), and vascular endothelial growth factor (VEGF) contribute to tissue remodeling and repair^22^. PRP has been widely employed in clinical practice for wound healing, musculoskeletal disorders, and soft-tissue regeneration due to its safety, accessibility, and ability to stimulate endogenous repair mechanisms^23^. In experimental SCI models, PRP facilitates neuronal survival, axonal regeneration, and recruitment of stem cells to the injury site^24^. When combined with biomaterials such as hydrogels, PRP provides synergistic benefits. The hydrogel serves as a structural scaffold and controlled-release matrix, while PRP delivers a sustained supply of bioactive molecules that enhance regeneration and functional recovery^25,26^.

Previous studies have investigated either PRP-based therapies or hydrogel scaffolds independently for neural regeneration. However, integrating PRP within a gelatin hydrogel matrix offers the potential to simultaneously provide structural support for injured spinal tissue and controlled release of multiple endogenous growth factors. This combined strategy may enhance the local regenerative microenvironment and improve functional recovery compared with approaches based solely on PRP delivery or hydrogel implantation.

This study therefore aims to develop and characterize a gelatin-based hydrogel incorporating PRP for SCI repair. The novelty of this work lies in the integration of platelet-derived bioactive factors within a gelatin scaffold to create a composite hydrogel capable of supporting neuronal viability while enabling sustained release of regenerative signaling molecules. We evaluate its biocompatibility, growth-factor release kinetics, and regenerative potential, focusing on its ability to promote neuronal proliferation, axonal extension, and functional restoration in vivo. Ultimately, this work seeks to determine whether this composite hydrogel system can enhance tissue regeneration and motor recovery following spinal cord injury.

## Methods

### Hydrogel preparation

A gelatin–PRP hydrogel was prepared by dissolving 5% (w/v) gelatin (type A, porcine skin, Sigma-Aldrich, St. Louis, MO, USA) in distilled water at 40–45 °C under continuous magnetic stirring until complete dissolution was achieved. The gelatin solution was subsequently crosslinked with 0.5% (w/v) glutaraldehyde (Sigma-Aldrich, USA) to form a stable gel matrix. Glutaraldehyde was selected as a crosslinking agent because it efficiently stabilizes gelatin networks through covalent bonding between amino groups, thereby improving hydrogel structural integrity and mechanical stability. Following crosslinking, the hydrogel was extensively washed three times with sterile phosphate-buffered saline (PBS) to minimize potential residual glutaraldehyde toxicity.Platelet-rich plasma (PRP) was isolated from male Sprague–Dawley rats (8–10 weeks old) using a two-step centrifugation protocol. Whole blood was first centrifuged at 200 × g for 10 min to separate erythrocytes from plasma. The supernatant was then centrifuged at 400 × g for 10 min to concentrate platelets. The platelet pellet was resuspended in a small volume of plasma to obtain PRP. PRP was freshly prepared for each independent experiment, and a separate rat was used for each PRP preparation.PRP was incorporated into the gelatin hydrogel at a 10% (v/v) concentration. The mixture was homogenized using gentle vortex mixing to ensure uniform distribution of PRP within the gelatin matrix and allowed to solidify at room temperature. The resulting hydrogel was stored at 4 °C prior to experimental use to preserve platelet-derived bioactive components.

### Material characterization

#### Scanning electron microscopy (SEM)

The morphological characteristics of the gelatin–PRP hydrogel were assessed using scanning electron microscopy (JEOL JSM-6510LV, JEOL Ltd., Tokyo, Japan). Hydrogel samples were first freeze-dried for 24 h and sputter-coated with a thin layer of gold prior to imaging. Microstructural images were obtained at magnifications ranging from 200 × to 2000 × to evaluate pore architecture and surface morphology. The average pore size and porosity were calculated using ImageJ software (National Institutes of Health, USA). The hydrogel displayed an average pore size of approximately 85 µm and a porosity of 78%, parameters considered suitable for cellular infiltration and nutrient diffusion.

#### Fourier transform infrared spectroscopy (FTIR)

Fourier transform infrared spectroscopy was performed using an FTIR spectrometer (Nicolet iS10, Thermo Fisher Scientific, USA) to analyze the chemical structure of the hydrogel and confirm successful PRP incorporation. Spectra were recorded in the range of 400–4000 cm⁻^1^ with a resolution of 4 cm⁻^1^. Characteristic peaks were observed at 1650 cm⁻^1^ (amide I), 1550 cm⁻^1^ (amide II), and 3300 cm⁻^1^ (amide A), confirming the presence of protein components in the hydrogel*.*

### In vitro* studies*

#### Cell viability (MTT assay)

Primary rat neuronal cells were cultured to evaluate hydrogel biocompatibility. Cells were seeded in 96-well plates at a density of approximately 1 × 10^4^ cells per well on hydrogel samples with a diameter of 5 mm and thickness of approximately 2 mm. Control cells consisted of neuronal cultures grown on standard tissue culture plates without hydrogel exposure. Neuronal morphology and growth characteristics were monitored using phase-contrast microscopy throughout the culture period. The majority of cells displayed characteristic neuronal morphology with extended neurites, indicating that the culture was predominantly composed of neuronal cells during the experimental period.Cell viability was assessed using the MTT assay at Days 1, 3, 5, and 7. MTT solution (0.5 mg/mL, Sigma-Aldrich, USA) was added to each well and incubated for 4 h at 37 °C. The resulting formazan crystals were dissolved in dimethyl sulfoxide (DMSO) and absorbance was measured at 570 nm using a microplate reader (BioTek ELx800, BioTek Instruments, USA). Cell viability was calculated as:

$${\mathrm{Cell}}\;{\mathrm{viability}}\left( \% \right) = \left( {{{{\mathrm{OD}}\;{\mathrm{of}}\;{\mathrm{treated}}\;{\mathrm{cells}}} \mathord{\left/ {\vphantom {{{\mathrm{OD}}\;{\mathrm{of}}\;{\mathrm{treated}}\;{\mathrm{cells}}} {{\mathrm{OD}}\;{\mathrm{of}}\;{\mathrm{control}}\;{\mathrm{cells}}}}} \right. \kern-0pt} {{\mathrm{OD}}\;{\mathrm{of}}\;{\mathrm{control}}\;{\mathrm{cells}}}}} \right) \times {1}00$$

Viability increased from 75% on Day 1 to 96% on Day 7, indicating strong compatibility between neuronal cells and the gelatin–PRP hydrogel.

### Growth factor release profile

The release kinetics of TGF-β1, PDGF-BB, and IGF-1 from the hydrogel were quantified using enzyme-linked immunosorbent assay (ELISA). Hydrogel samples were incubated in phosphate-buffered saline (PBS, pH 7.4) at 37 °C, and aliquots of the supernatant were collected at 0, 6, 12, 24, 48, 72, and 96 h.Growth factor concentrations were measured using commercial ELISA kits (R&D Systems, Minneapolis, MN, USA) following the manufacturer’s instructions. Absorbance was measured using a microplate reader (BioTek ELx800, USA), and concentrations were determined based on standard calibration curves provided with each kit.Growth factor release values were expressed as cumulative percentages of the total growth factor content measured in the PRP preparation prior to incorporation into the hydrogel*.*The release profile demonstrated a biphasic pattern consisting of an initial burst release during the first 12 h followed by a sustained release phase from 24 to 96 h, with 85–88% of the total growth factors released by 96 h.

### In vivo studies

#### Animal model and surgical procedure

Male Sprague–Dawley rats (8–10 weeks old, weighing 220–250 g) were obtained from the institutional animal facility of Islamic Azad University, Karaj Branch, Iran. All experimental procedures were conducted in accordance with institutional animal care guidelines.Animals were anesthetized using ketamine (80 mg/kg) and xylazine (10 mg/kg) administered intraperitoneally. After anesthesia, a laminectomy was performed at the thoracic level T9–T10 to expose the spinal cord. A moderate contusion injury was induced using a spinal cord impactor in which a 10 g rod was dropped from a height of approximately 25 mm onto the exposed spinal cord segment.

Animals were randomly divided into four groups (n = 6 animals per group):

1. Negative control (SCI without treatment)
2. Hydrogel treatment
3. PRP treatment
4. PRP + Hydrogel composite treatment

**Fig. 1:**
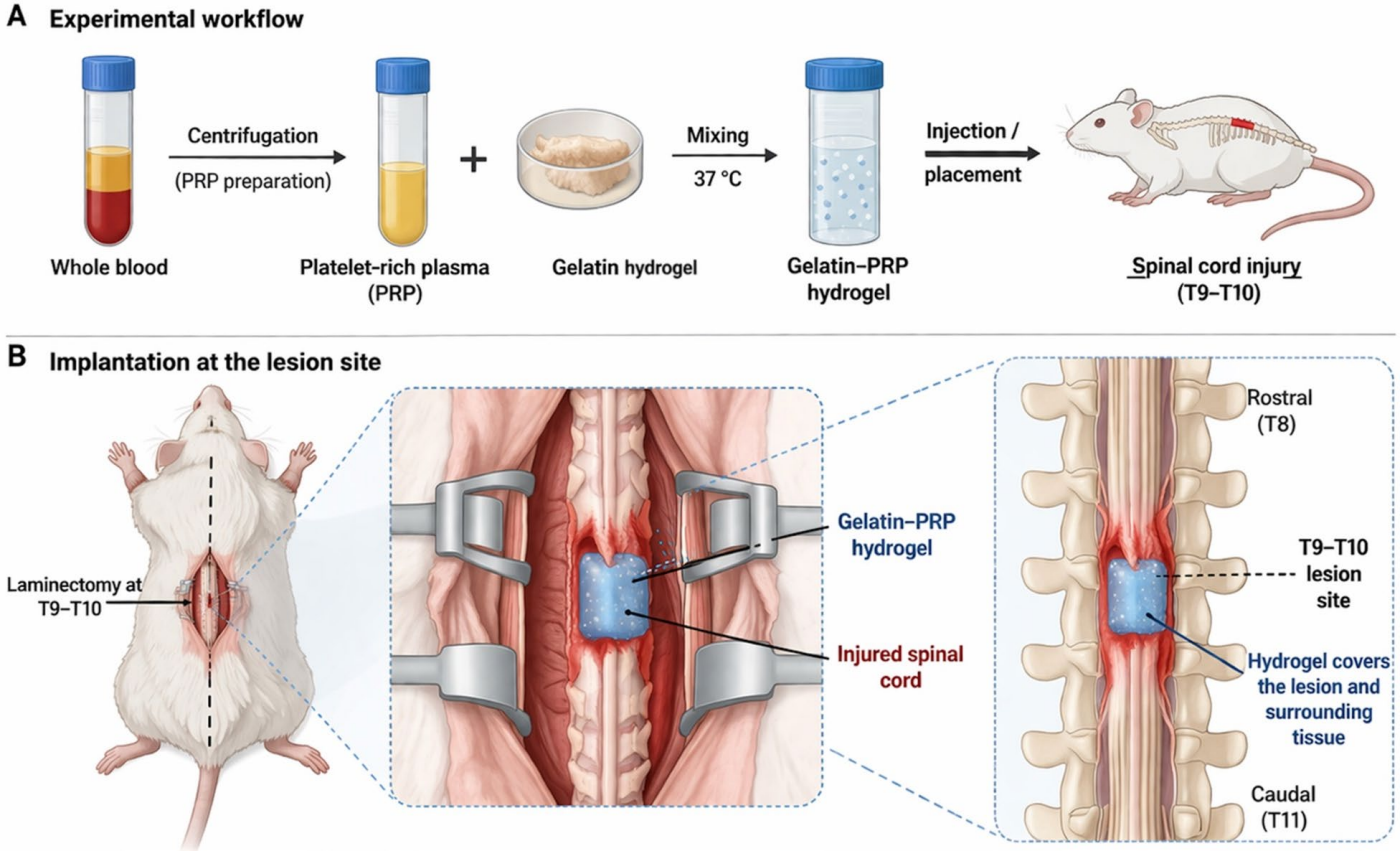
Schematic representation of the preparation and implantation of the gelatin–PRP hydrogel in a spinal cord injury model. (**A**) Preparation of platelet-rich plasma (PRP) from whole blood followed by incorporation into gelatin to form the composite hydrogel. (**B**) Surgical exposure of the spinal cord at the T9–T10 level and direct application of the hydrogel at the lesion site, ensuring coverage of the injured tissue.

In the PRP-only group, freshly prepared PRP was directly applied onto the exposed spinal cord lesion immediately after injury induction to ensure local delivery of platelet-derived growth factors. In the hydrogel group, gelatin hydrogel was placed locally onto the injured spinal cord surface. In the combined treatment group, PRP-loaded gelatin hydrogel was applied directly onto the lesion area to cover the injured tissue. Thus, the application site was the lesion site itself, not the surrounding intact tissue. Following surgery, muscle and skin were sutured using sterile absorbable sutures, and animals received postoperative care including subcutaneous saline for hydration and manual bladder expression twice daily until recovery of bladder function. A schematic illustration of the hydrogel implantation procedure is provided in Fig. 1.

### Functional recovery assessment

Motor function recovery was evaluated weekly for 4 weeks using the Basso, Beattie, and Bresnahan (BBB) locomotor scale, which assesses hindlimb movement and coordination during open-field locomotion.Animals were placed individually in an open-field arena measuring approximately 90 cm × 90 cm, and their movements were observed for 4 min. BBB scoring was performed by two independent observers blinded to treatment allocation to minimize subjective bias.

### Histological analysis

At the end of the experimental period, animals were deeply anesthetized and transcardially perfused with phosphate-buffered saline followed by 4% paraformaldehyde fixation solution. Spinal cord segments containing the lesion site were harvested, post-fixed in 4% paraformaldehyde, and embedded in paraffin.Tissue sections (5 µm thickness) were prepared using a microtome and stained with hematoxylin and eosin (H&E) to assess tissue morphology, neuronal survival, and lesion architecture. Histological scoring was performed by a blinded pathologist to evaluate neuronal preservation, tissue damage, and cavity formation at the injury site. Lesion cavity size was quantified using ImageJ software by measuring the lesion area in serial sections of the injured spinal cord.

### Statistical analysis

All data are expressed as mean ± standard deviation (SD). Statistical analyses were performed using two-way analysis of variance (ANOVA) followed by Tukey’s post hoc test for pairwise comparisons. A *p*-value < 0.05 was considered statistically significant. Statistical analysis was conducted using SPSS software (version 24.0, IBM Corp., USA).

## Results

### Material characterization

#### Scanning electron microscopy (SEM)

**Fig. 2:**
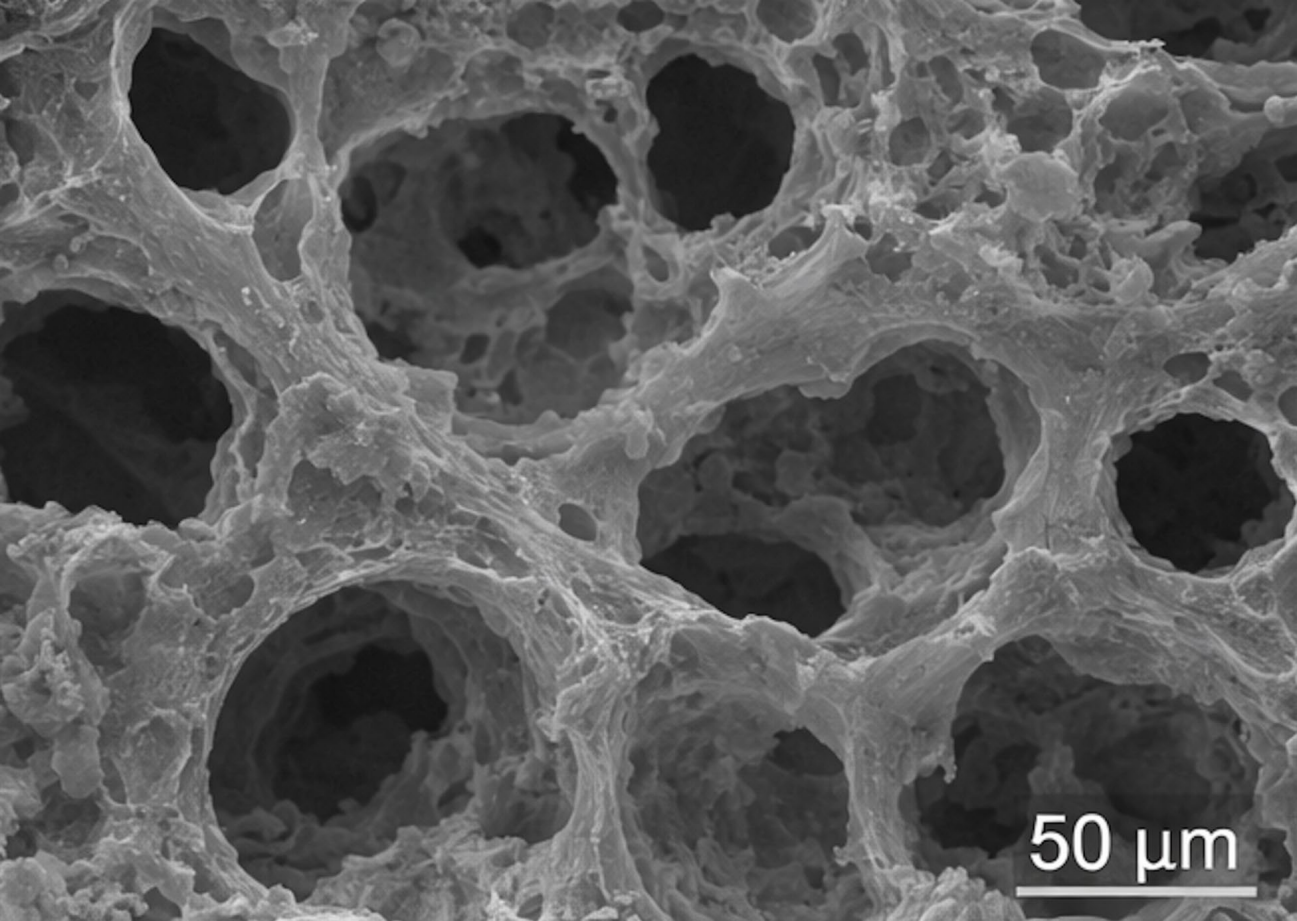
Scanning electron microscopy (SEM) image of the gelatin–platelet-rich plasma (PRP) hydrogel showing a highly porous and interconnected microstructure. The hydrogel exhibited an average pore size of approximately 85 µm and a porosity of 78%, providing a favorable environment for cellular infiltration and nutrient diffusion. Scale bar: 50 µm.

Scanning electron microscopy revealed that the gelatin–PRP hydrogel possessed a highly porous and interconnected microstructure (Fig. 2). The pores were uniformly distributed throughout the scaffold and formed a three-dimensional network suitable for cellular infiltration and nutrient transport. Quantitative analysis using ImageJ software indicated an average pore diameter of approximately 85 ± 6 µm, with an overall porosity of approximately 78%.These structural features are considered favorable for neural tissue engineering because interconnected pores facilitate cell migration, oxygen diffusion, and nutrient exchange, which are essential for maintaining cellular viability and supporting tissue regeneration following spinal cord injury.

#### Fourier transform infrared spectroscopy (FTIR)

**Fig. 3:**
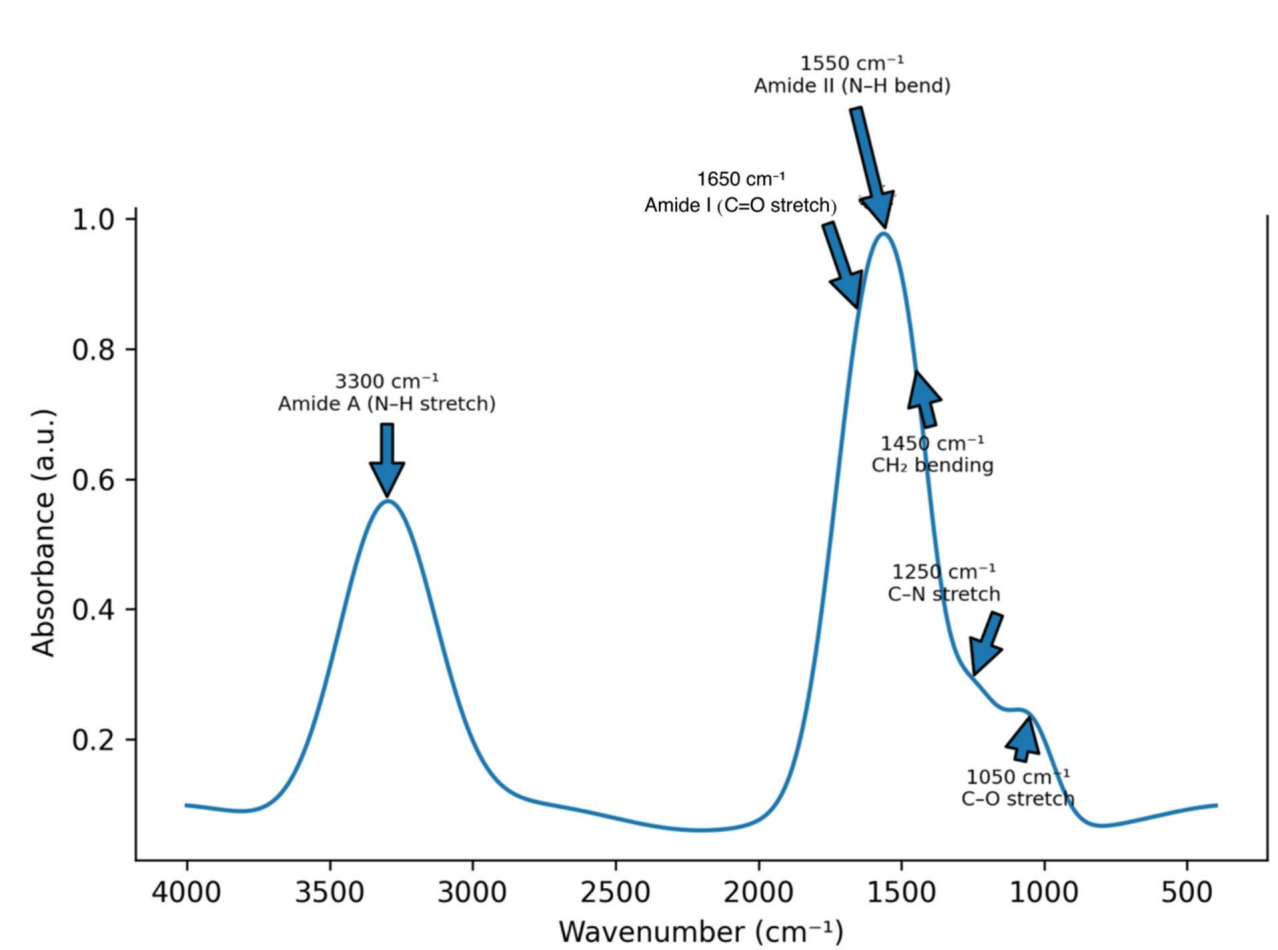
Fourier transform infrared (FTIR) spectrum of the gelatin–platelet-rich plasma (PRP) hydrogel. Characteristic absorption peaks were observed at 3300 cm⁻^1^ (amide A, N–H stretching vibration), 1650 cm⁻^1^ (amide I, C=O stretching vibration), and 1550 cm⁻^1^ (amide II, N–H bending vibration), confirming the presence of protein functional groups associated with gelatin and PRP components. Additional peaks at 1450 cm⁻^1^ (CH₂ bending vibration), 1250 cm^−1^ (C–N stretching vibration), and 1050 cm⁻^1^ (C–O stretching vibration) further indicate the presence of protein and polymer functional groups within the gelatin–PRP hydrogel matrix.

FTIR analysis confirmed the successful incorporation of platelet-rich plasma into the gelatin matrix (Fig. 3). The FTIR spectrum exhibited several characteristic absorption peaks corresponding to protein and polymer functional groups.A broad peak at 3300 cm⁻^1^ corresponded to N–H stretching vibrations (amide A) associated with protein structures. The prominent band observed at 1650 cm⁻^1^ was attributed to C=O stretching vibrations (amide I), while the peak at 1550 cm⁻^1^ corresponded to N–H bending vibrations (amide II).Additional peaks were detected at 1450 cm⁻^1^ (CH₂ bending), 1250 cm⁻^1^ (C–N stretching), and 1050 cm⁻^1^ (C–O stretching), further confirming the presence of both gelatin polymer chains and PRP-derived protein components within the composite hydrogel. These spectral characteristics confirm the presence of both gelatin and PRP-derived protein components within the composite hydrogel (Table 1)*.*

**Table 1:** FTIR spectral peaks and corresponding functional groups identified in the gelatin–PRP hydrogel.

Wavenumber (cm⁻^1^)	Functional group assignment
3300	N–H stretching vibration (Amide A)
1650	C=O stretching vibration (Amide I)
1550	N–H bending vibration (Amide II)
1450	CH₂ bending vibration
1250	C–N stretching vibration
1050	C–O stretching vibration

FTIR analysis was performed using a Nicolet iS10 Fourier transform infrared spectrometer (Thermo Fisher Scientific, USA). Peaks correspond to characteristic vibrational bands associated with gelatin and protein components present in platelet-rich plasma. FTIR, Fourier transform infrared spectroscopy; PRP, Platelet-rich plasma.

### In vitro* evaluation*

#### Cell viability (MTT assay)

The biocompatibility of the gelatin–PRP hydrogel was assessed using an MTT assay performed on primary rat neuronal cultures. Cells were seeded on hydrogel discs and monitored over a 7-day culture period.Cell viability progressively increased throughout the experiment (Table 2). Viability values increased from 75 ± 4% on Day 1 to 85 ± 3% on Day 3, 92 ± 2% on Day 5, and 96 ± 2% on Day 7 (n = 6, *p* < 0.05).These results demonstrate that the gelatin–PRP hydrogel provides a supportive microenvironment for neuronal survival and proliferation. The continuous increase in metabolic activity suggests that the hydrogel scaffold not only supports cell adhesion but also promotes sustained neuronal viability.

**Table 2:** Cell viability of neuronal cells cultured on gelatin–PRP hydrogel evaluated by MTT assay.

Day	Cell viability (%) (mean ± SD)	n
1	75 ± 4	6
3	85 ± 3	6
5	92 ± 2	6
7	96 ± 2	6

Cell viability was determined using the MTT assay and calculated as (OD of treated cells / OD of control cells) × 100. Control cells consisted of neuronal cultures grown on standard tissue culture plates without hydrogel exposure. Data are presented as mean ± SD from six independent replicates. PRP, Platelet-rich plasma; MTT, 3-(4,5-dimethylthiazol-2-yl)-2,5-diphenyltetrazolium bromide; SD, Standard deviation.

### Growth factor release profile

**Fig. 4:**
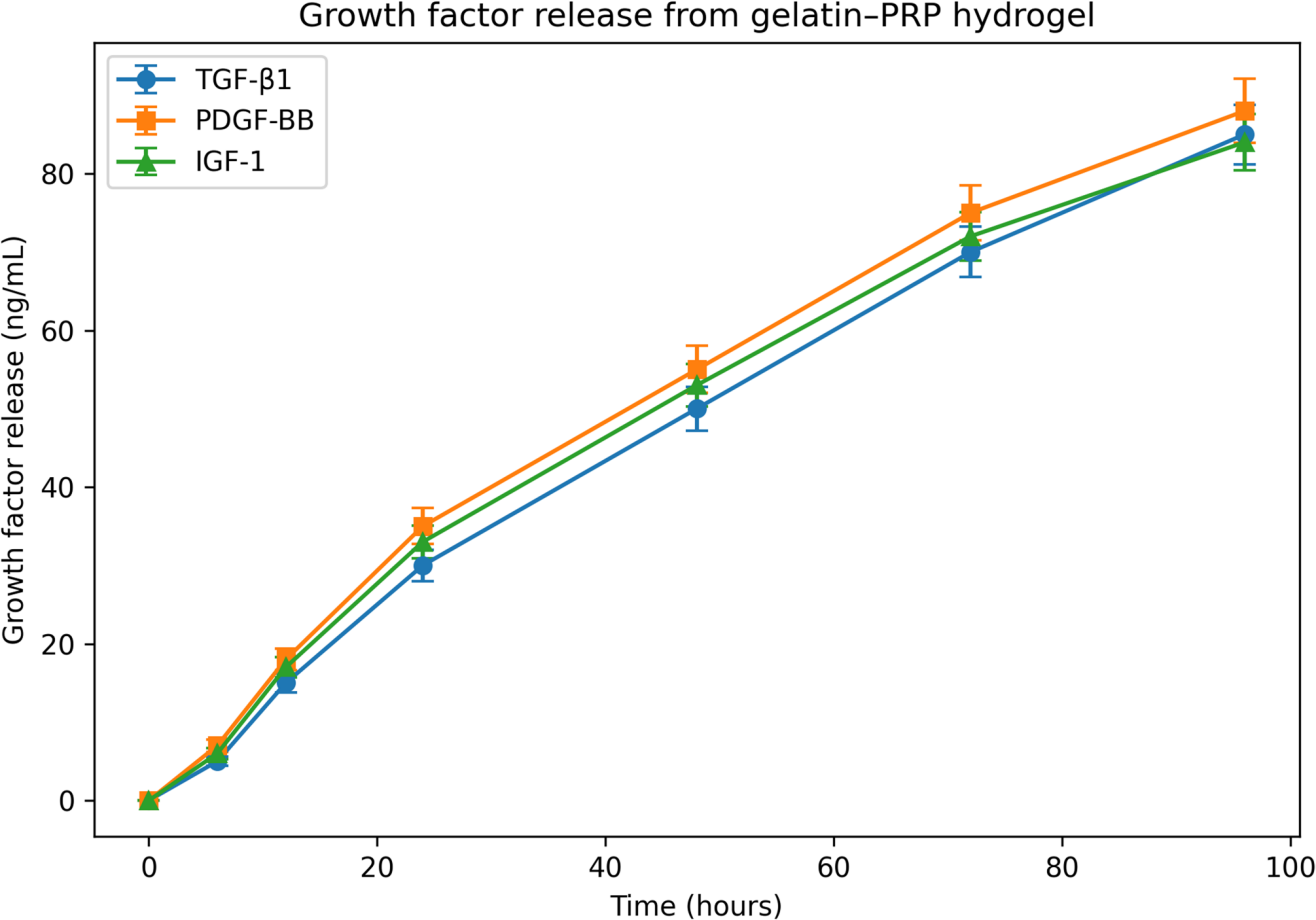
Growth factor release profile of the gelatin–PRP hydrogel over 96 h. The release of TGF-β1, PDGF-BB, and IGF-1 followed a biphasic pattern, with an initial burst release during the first 12 h followed by sustained release up to 96 h (*p* < 0.05). Data are presented as mean ± SD (n = 6 independent measurements).

The release kinetics of key growth factors from the gelatin–PRP hydrogel were evaluated over a 96-h period using ELISA assays (Fig. 4; Table 3). The hydrogel exhibited a biphasic release pattern.During the initial phase, a rapid burst release occurred within the first 12 h, corresponding to approximately 10–20% of total growth factor release. This was followed by a sustained release phase between 24 and 96 h, during which growth factors were gradually released from the hydrogel matrix.By the end of the observation period, cumulative release reached 85 ± 3.8 ng/mL for TGF-β1, 88 ± 4.1 ng/mL for PDGF-BB, and 84 ± 3.6 ng/mL for IGF-1 (n = 6, *p* < 0.05). The release profile showed that 85–88% of the initial growth factor content (measured prior to hydrogel incorporation) was detected in the incubation buffer by 96 h*.*

**Table 3:** Growth factor release profile from gelatin–PRP hydrogel over 96 h.

Time (hours)	TGF-β1 (ng/mL) (mean ± SD)	PDGF-BB (ng/mL) (mean ± SD)	IGF-1 (ng/mL) (mean ± SD)	n
0	0 ± 0	0 ± 0	0 ± 0	6
6	5 ± 0.6	7 ± 0.8	6 ± 0.7	6
12	15 ± 1.2	18 ± 1.4	17 ± 1.3	6
24	30 ± 2.0	35 ± 2.3	33 ± 2.1	6
48	50 ± 2.8	55 ± 3.0	53 ± 2.7	6
72	70 ± 3.2	75 ± 3.5	72 ± 3.1	6
96	85 ± 3.8	88 ± 4.1	84 ± 3.6	6

Growth factor concentrations were quantified using ELISA kits (R&D Systems, USA). Values represent cumulative release of growth factors from the gelatin–PRP hydrogel during incubation in PBS at 37 °C. Data are expressed as mean ± SD from six independent measurements. TGF-β1, Transforming growth factor beta-1; PDGF-BB, Platelet-derived growth factor-BB; IGF-1, Insulin-like growth factor-1; PRP, Platelet-rich plasma; SD, Standard deviation; ELISA, Enzyme-linked immunosorbent assay.

### Functional recovery assessment

**Fig. 5:**
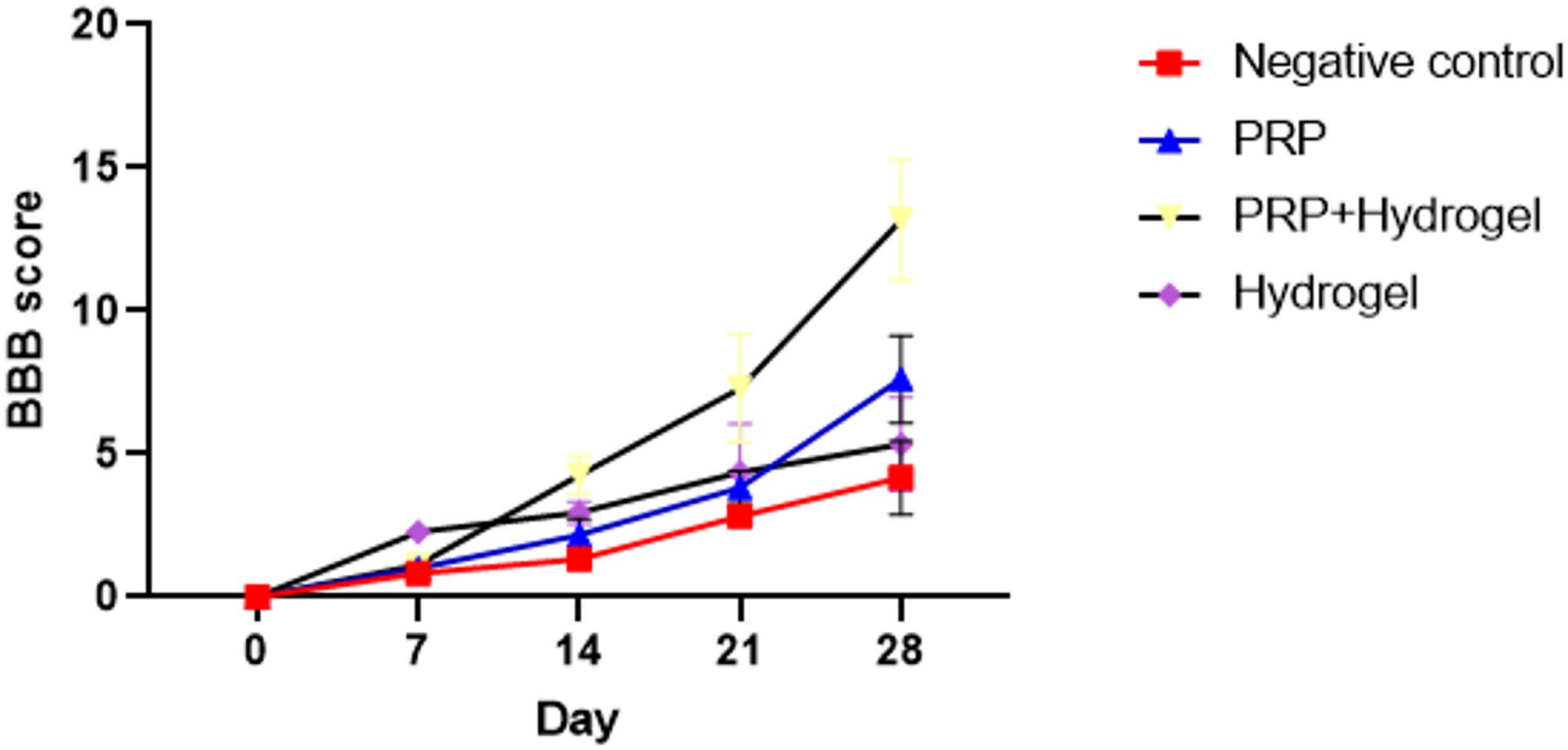
Basso–Beattie–Bresnahan (BBB) locomotor scores of rats in different treatment groups (Negative Control, Hydrogel, PRP, PRP + Hydrogel) during the 28-day post-injury period. The PRP + Hydrogel group exhibited significantly greater functional recovery compared with other groups (*p* < 0.05). Data are presented as mean ± SD (n = 6 animals per group).

Functional recovery following spinal cord injury was evaluated using the Basso, Beattie, and Bresnahan (BBB) locomotor scoring system over a 28-day observation period (Fig. 5).At the initial time point (Day 0), all animals exhibited a BBB score of zero, indicating complete hindlimb paralysis following injury. Progressive improvement in locomotor function was observed in all treatment groups over time; however, the magnitude of recovery differed significantly among groups (*p* < 0.05).The PRP + Hydrogel group demonstrated the greatest functional improvement, with BBB scores increasing steadily from 2.8 ± 0.6 at Day 7 to 13.2 ± 2.0 at Day 28. In contrast, the PRP group reached a score of 7.8 ± 1.2, while the Hydrogel group achieved 5.4 ± 0.9 at the same time point. The Negative Control group exhibited minimal recovery, with scores remaining below 4.0 ± 1.0 throughout the experiment.Statistical analysis confirmed that locomotor scores in the PRP + Hydrogel group were significantly higher than those of all other groups from Day 14 onward (*p* < 0.05). These findings indicate that the combined treatment significantly enhances motor recovery following spinal cord injury.

### Histopathological findings

**Fig. 6:**
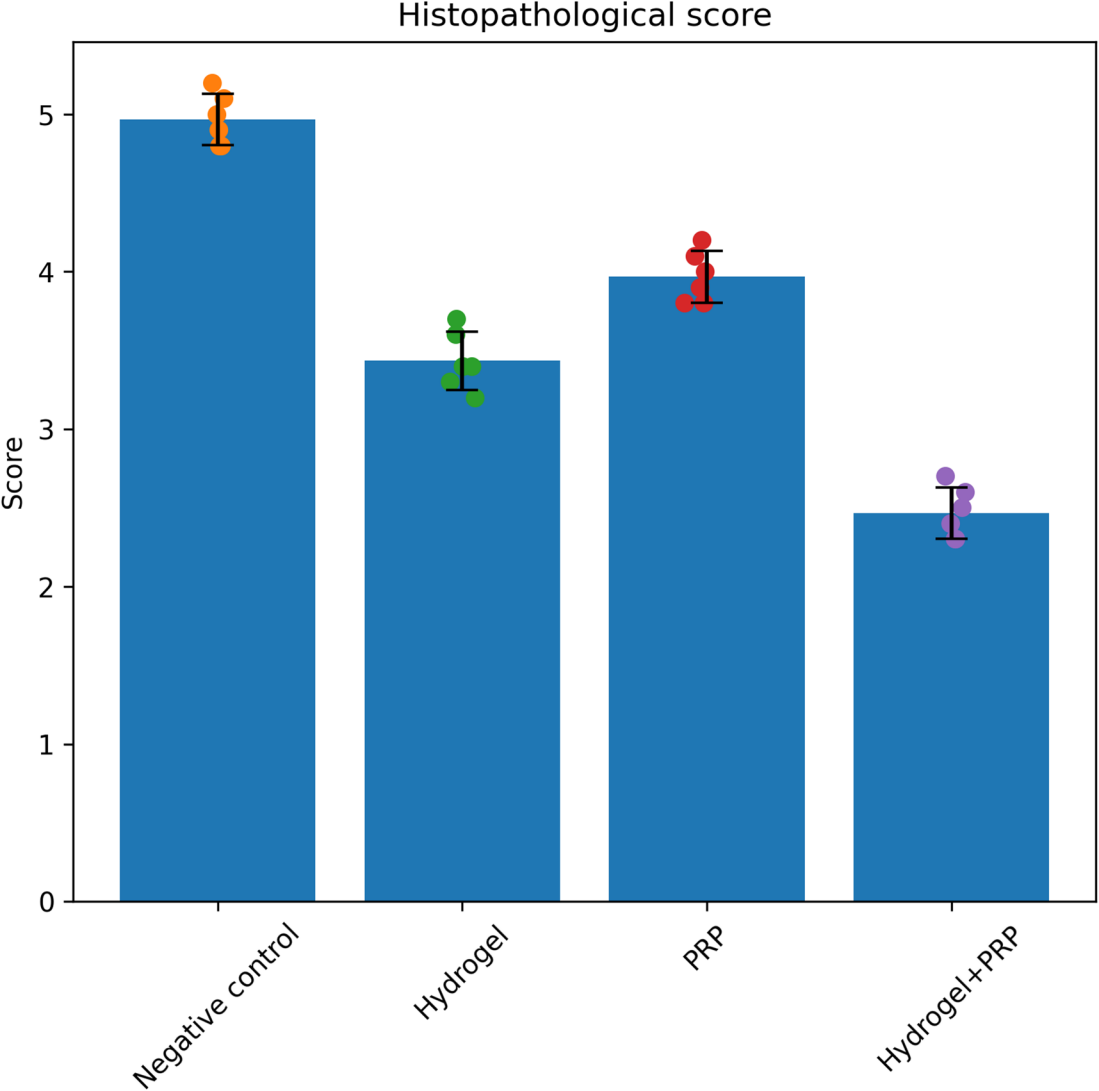
Histological scoring of spinal cord tissue 28 days after injury in different treatment groups (Negative Control, Hydrogel, PRP, PRP + Hydrogel). The PRP + Hydrogel group demonstrated significantly lower tissue damage and greater neuronal preservation compared with other groups. Data are presented as mean ± SD (n = 6 animals per group).

**Fig. 7:**
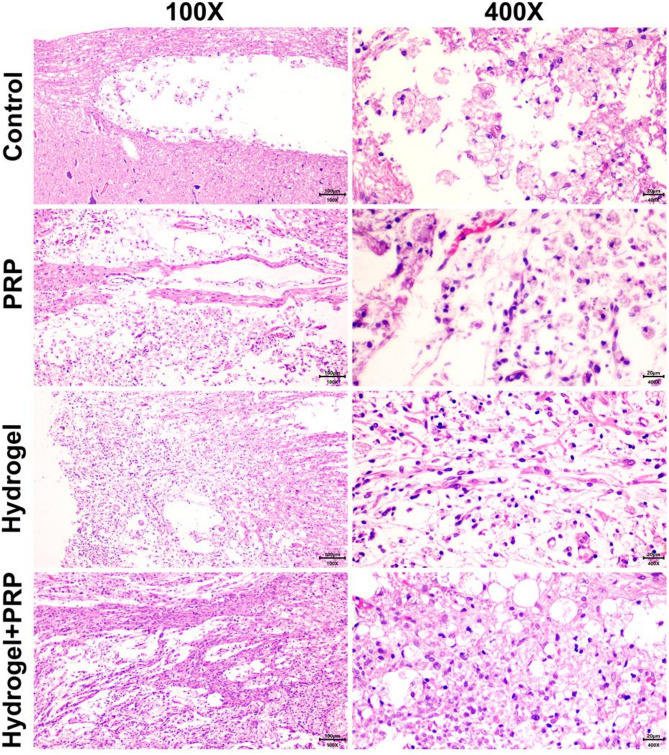
Representative histopathological images of spinal cord tissue 28 days after injury under different treatment conditions (Negative Control, Hydrogel, PRP, PRP + Hydrogel). Hematoxylin and eosin (H&E) stained sections are shown at 100 × (left) and 400 × (right) magnifications. The PRP + Hydrogel group shows reduced lesion cavity formation and improved neuronal preservation compared with other groups. Scale bars: 100 μm (100 ×) and 20 μm (400 ×).

Histological analysis was performed 28 days after injury to evaluate tissue preservation and cellular responses at the lesion site (Figs. 6 and 7). Quantitative histopathological scoring demonstrated significant differences among the experimental groups (*p* < 0.05).The Negative Control group exhibited extensive pathological changes, including large cystic cavities, widespread necrosis, and dense scar-like tissue formation. These structural abnormalities are characteristic of untreated spinal cord injury.In the Hydrogel group, moderate tissue preservation was observed. Although neuronal loss and glial scarring were present, the lesion cavity appeared smaller compared with the control group.The PRP treatment group showed improved tissue architecture relative to the control group, with reduced necrosis and moderate neuronal survival. PRP treatment also promoted increased cellular infiltration, including glial and macrophage populations involved in tissue repair. The PRP + Hydrogel group exhibited the most favorable histological outcome. Tissue sections demonstrated significantly reduced lesion cavities compared with all other groups (*p* < 0.05). Improved tissue preservation and reduced scar-like appearance were also observed. Moderate axonal regeneration and organized cellular infiltration were also evident.Quantitative histological scoring further confirmed these observations, with the PRP + Hydrogel group displaying the lowest tissue damage score and the highest neuronal preservation among all experimental groups (Fig. 6; n = 6 animals per group).

## Discussion

This study demonstrates the therapeutic potential of a gelatin–PRP hydrogel for spinal cord injury (SCI) repair. In vitro analyses confirmed excellent biocompatibility, as shown by a progressive increase in neuronal cell viability from 75% on Day 1 to 96% on Day 7 (*p* < 0.05). The hydrogel also released bioactive factors in a biphasic pattern, with 85–88% of TGF-β1, PDGF-BB, and IGF-1 detected in the incubation buffer within 96 h (*p* < 0.05). This release profile suggests that growth factors are released from the gelatin matrix over time, which may contribute to the local microenvironment during the early stages of neural repair. In vivo, the PRP + Hydrogel group exhibited the least tissue damage, reduced scar-like tissue appearance, improved tissue preservation, and better histological outcomes compared with other treatment groups*.* These findings collectively indicate that combining a structural scaffold with platelet-derived bioactive factors may create a supportive microenvironment that promotes neural tissue preservation and functional improvement following SCI.

The results are consistent with previous reports emphasizing the benefits of PRP-based and hydrogel-based biomaterials in neural regeneration. Prior investigations have shown that hydrogels provide a hydrated extracellular matrix like microenvironment that facilitates axonal regrowth and limits glial scar formation^7,13–16^. Similarly, PRP has been demonstrated to supply multiple growth factors including TGF-β, PDGF, and VEGF that promote angiogenesis, cellular proliferation, and neuronal survival^21–23^. In the present study, the gelatin scaffold served not only as a structural support but also as a reservoir for sustained release of PRP-derived growth factors, thereby enhancing the local regenerative microenvironment at the injury site. Our findings expand on this knowledge by showing that a gelatin matrix can serve as an effective carrier for PRP, providing both structural support and controlled molecular delivery over a defined temporal window, thus maximizing the regenerative response.

Previous studies combining stem-cell-based therapies or PRP within chitosan or hydrogel scaffolds have also reported enhanced angiogenesis and tissue repair^9,19,20^. While those studies focused primarily on stem cell mediated regeneration, the present work provides quantitative confirmation of enhanced neuronal viability, showing a 21% increase over seven days (*p* < 0.05). Salarinia et al. observed improved motor function in PRP-treated rats, with significant gains in Basso Beattie Bresnahan (BBB) scores by Week 5^24^. Consistently, our PRP + Hydrogel group exhibited steady improvement in body condition and locomotor function throughout the 28 day evaluation period. Histological findings in both studies revealed reduced glial scarring and greater axonal regeneration, reinforcing the role of PRP in promoting functional recovery. More recently, Nie et al. demonstrated that PRP-derived exosomes stabilize the blood spinal cord barrier and reduce neuroinflammation, thereby enhancing recovery in SCI^26^. Although their approach employed exosome mediated delivery rather than a structural scaffold, both strategies converge on the concept of harnessing PRP intrinsic regenerative signaling.

The biphasic growth factor release kinetics observed in this study are of particular significance. An initial burst release supports the rapid recruitment of reparative and immune cells to the injury site, while the subsequent sustained phase maintains a pro regenerative environment for neurogenesis, angiogenesis, and axonal extension^14,17,18^. Controlled delivery systems such as hydrogels are known to preserve growth factor stability and concentration gradients, which are crucial for effective neural repair^13–16^. The ability of the gelatin PRP composite to regulate this temporal release likely underpins its therapeutic efficacy.

Histopathological evaluation further corroborated these functional outcomes. The PRP + Hydrogel group exhibited minimal tissue disruption and reduced cavity formation compared with all other groups. These outcomes align with previous reports showing that hydrogel scaffolds and PRP-derived factors synergistically enhance tissue preservation and limit fibrotic scar formation^10,15,23,25^. Although hematoxylin and eosin staining provided valuable information regarding tissue morphology, lesion cavity formation, and cellular infiltration, this method does not allow precise identification of specific neural cell populations. Future studies incorporating immunohistochemical markers such as NeuN for neurons, GFAP for astrocytes, and Iba-1 for microglia will be necessary to definitively assess glial scar density and neuronal survival.

Functional recovery in this study was evaluated using the BBB locomotor scoring system, which is widely used for assessing hindlimb motor function in rodent models of SCI^24^. While BBB scoring provides valuable functional information, it relies partly on observational assessment and animal motivation. Additional behavioral tests such as ladder climbing or grid walking assays could further improve the robustness of functional evaluation in future studies.

Despite these promising results, certain limitations must be acknowledged. The 28-day follow-up may not fully capture the long-term regenerative capacity of the gelatin–PRP hydrogel, and extended assessments are necessary. Additionally, while the rat contusion model is widely accepted, it does not entirely reproduce the complexity of human SCI pathophysiology, and large animal studies will be essential for translation. Growth factor release was quantified only in the incubation buffer; residual growth factors within the hydrogel after washing were not measured. Histological assessments were performed using H&E staining only; immunohistochemical markers for glial scars (GFAP), neurons (NeuN), and microglia/macrophages (Iba-1) were not included. FTIR analysis confirmed the presence of protein components but did not demonstrate specific molecular interactions between gelatin and PRP proteins. Additional molecular analyses (qPCR, Western blotting, flow cytometry) are needed to elucidate underlying mechanisms, and optimizing hydrogel composition will be critical for future applications.

## Conclusion

This study demonstrates that gelatin–PRP hydrogels may represent a promising biomaterial-based strategy for spinal cord injury (SCI) repair. The hydrogel supported robust cell proliferation and released key growth factors (TGF-β1, PDGF-BB, IGF-1) in a biphasic pattern over 96 h, and promoted neuronal survival, axonal regeneration, and functional recovery in SCI models. Among all treatment groups, the PRP + Hydrogel combination achieved the least tissue damage and the greatest motor recovery, underscoring the synergistic benefits of integrating PRP within a gelatin scaffold. However, additional mechanistic studies and long-term in vivo investigations are necessary to fully elucidate the regenerative pathways involved and to confirm the translational potential of this biomaterial system. Nonetheless, the results contribute valuable evidence supporting biomaterial-based therapies for SCI and lay a foundation for future innovations in regenerative medicine.

## Data Availability

All data generated or analyzed during this study are included in this published article. Additional datasets are available from the corresponding author (H.-U.H.) upon reasonable request.
